# Combined therapeutic effects of Renshenguben and swimming on knee osteoarthritis associated with the gut-joint axis and HDAC3/PPAR-*γ* signaling pathway

**DOI:** 10.3389/fmicb.2026.1930569

**Published:** 2026-09-08

**Authors:** Ziqiang Huang, Zhenhong He, Mei Yang, Xiaohua Jiang, Ningchuan Zhang, Dingsu Bao

**Affiliations:** 1 Ziyang City People's Hospital, Ziyang, Sichuan, China; 2 Department of Orthopedics, The Affiliated Traditional Chinese Medicine Hospital, Southwest Medical University, Luzhou, Sichuan, China

**Keywords:** gut-joint axis, HDAC3, knee osteoarthritis, PPAR-γ, Renshenguben, swimming

## Abstract

**Background:**

Knee osteoarthritis (KOA) is a chronic degenerative joint disease characterized by cartilage destruction, subchondral bone remodeling, and inflammation. Gut microbiota dysbiosis and aberrant HDAC3/PPAR-γ signaling contribute to KOA progression. This study investigated the therapeutic effects of Renshenguben (RSGB) combined with swimming exercise in monosodium iodoacetate (MIA)-induced KOA and explored the underlying mechanisms.

**Methods:**

Forty-five male Sprague–Dawley (SD) rats were randomly divided into Control, MIA, RSGB, Swimming, and Swimming+RSGB groups. KOA was induced by intra-articular MIA injection. RSGB was administered orally, and swimming exercise was performed for 4 weeks. Knee swelling, mechanical pain threshold, and motor performance were assessed. Serum inflammatory cytokines, cartilage degradation markers, and gut barrier indicators were measured by ELISA. Cartilage histology, immunohistochemistry, micro-computed tomography, qPCR, and western blot analyses were performed to evaluate joint pathology and HDAC3/PPAR-γ expression. Gut microbiota composition was analyzed using 16S rRNA gene sequencing.

**Results:**

MIA induced joint inflammation, pain hypersensitivity, motor dysfunction, elevated CRP, COMP, MMP-1, MMP-13, TNF-α, IL-1β, NF-κB, DAO, and LPS, and decreased IL-10. RSGB or swimming alone partially alleviated these changes, while combined treatment showed the most pronounced improvements. Combined treatment preserved cartilage and subchondral bone, suppressed HDAC3, restored PPAR-γ expression. Additionally, KOA-associated gut dysbiosis was characterized by reduced microbial diversity and depletion of SCFA-producing bacteria, which was significantly reversed by combined treatment.

**Conclusion:**

RSGB combined with swimming exerts enhanced protective effects in KOA by reducing inflammation, improving gut barrier function, restoring microbiota homeostasis, and regulating the HDAC3/PPAR-*γ* axis, offering a promising integrative therapeutic strategy.

## Highlights

- **Findings:** RSGB combined with swimming produced beneficial therapeutic effects in MIA-induced knee osteoarthritis rats, significantly improving pain behavior, joint swelling, motor function, and cartilage/subchondral bone integrity. The combined intervention also reduced systemic inflammation, restored intestinal barrier function, modulated gut microbiota dysbiosis, and normalized the HDAC3/PPAR-γ signaling axis.
- **Implications:** These results suggest that integrating pharmacological treatment with structured exercise may offer superior benefits over monotherapy in KOA by targeting both systemic inflammation and the gut–joint axis. The study provides experimental evidence supporting HDAC3/PPAR-γ as a key mechanistic link between microbiota-derived metabolites and joint degeneration, highlighting a potential multi-target therapeutic strategy for osteoarthritis management.
- **Caution:** This study is based on a monosodium iodoacetate-induced rat model, which may not fully replicate the complexity and heterogeneity of human knee osteoarthritis. The translational relevance to clinical patients remains limited, and long-term efficacy, safety, and optimal dosing/exercise parameters were not evaluated.

## Introduction

Knee osteoarthritis (KOA) is a chronic degenerative joint disease characterized by progressive cartilage degeneration, synovial inflammation, subchondral bone remodeling, and osteophyte formation (Elmajee et al., 2025). As one of the most common musculoskeletal disorders among the elderly population, KOA has become a major public health burden worldwide due to its high prevalence, disability rate, and substantial socioeconomic costs (Kim, 2008; Cross et al., 2014; Silverwood et al., 2015; Chung et al., 2016; Loza et al., 2009). Patients with KOA frequently suffer from persistent joint pain, stiffness, swelling, and impaired mobility, which severely reduce quality of life and physical function (Wojcieszek et al., 2022). With the accelerating global aging process and the increasing prevalence of obesity and metabolic disorders, the incidence of KOA continues to rise annually (Shumnalieva et al., 2023; Song et al., 2024). Although current therapeutic strategies, including nonsteroidal anti-inflammatory drugs (NSAIDs), intra-articular injections, physical rehabilitation, and joint replacement surgery, can partially relieve symptoms, these approaches are mainly focused on symptomatic management and are unable to effectively halt disease progression or promote cartilage regeneration (Ghouri and Conaghan, 2020; Xu et al., 2021). Therefore, identifying novel therapeutic targets and developing comprehensive intervention strategies for KOA remain urgent clinical challenges.

Traditionally, KOA was considered a “wear-and-tear” disease caused primarily by mechanical degeneration (Chan et al., 2021). However, accumulating evidence indicates that KOA is a multifactorial disorder involving chronic low-grade inflammation, immune dysregulation, oxidative stress, metabolic abnormalities, and extracellular matrix degradation (Thomas et al., 2018; Batushansky et al., 2022; Zhu et al., 2018). Pro-inflammatory cytokines such as tumor necrosis factor-α (TNF-α) and interleukin-1β (IL-1β) activate nuclear factor kappa-B (NF-κB) signaling and subsequently induce the expression of matrix metalloproteinases (MMPs), including MMP1 and MMP13, leading to cartilage extracellular matrix degradation and chondrocyte apoptosis (Zhou et al., 2024; Park et al., 2021; Han et al., 2021). Increasing evidence has highlighted the critical involvement of epigenetic dysregulation in the pathogenesis of KOA. Among these regulators, histone deacetylase 3 (HDAC3) has attracted considerable attention due to its abnormal activation in osteoarthritic cartilage. Previous studies have shown that elevated HDAC3 expression is associated with enhanced inflammatory responses and increased catabolic activity in chondrocytes, particularly through promoting IL-1β-induced MMP expression and extracellular matrix degradation (Xu et al., 2021; Meng et al., 2018). In contrast, peroxisome proliferator-activated receptor-γ (PPAR-γ) is recognized as an important protective factor involved in maintaining cartilage homeostasis and suppressing inflammatory signaling (Li et al., 2014; Zhu et al., 2019; Saber et al., 2023). Compared with wild-type mice, PPAR-γ-deficient mice exhibit aggravated OA progression (Vasheghani et al., 2013; Vasheghani et al., 2015; Setoguchi et al., 2001). While activation of PPAR-γ by activators exerts significant anti-inflammatory and chondroprotective effects, thereby attenuating OA development and progression (Kobayashi et al., 2005; Boileau et al., 2007). These findings suggest that PPAR-γ may serve as a promising therapeutic target for OA intervention. Notably, HDAC3 and PPAR-γ may exhibit a potential functional association in KOA. Previous studies have suggested that PPAR-γ may exert anti-inflammatory effects through functional interaction with HDAC3-associated corepressor complexes, indicating a potential regulatory crosstalk between PPAR-γ and HDAC3 during inflammatory responses (Pascual et al., 2005). Therefore, the HDAC3/PPAR-γ signaling axis has gradually emerged as a pivotal molecular pathway in the pathogenesis of KOA.

Recently, the concept of the “gut-joint axis” has attracted considerable attention in osteoarthritis research. The intestinal tract is not only a digestive organ but also the largest immune organ in the human body (Vighi et al., 2008). Disruption of intestinal barrier integrity and gut microbiota dysbiosis can increase intestinal permeability, allowing harmful bacterial metabolites such as lipopolysaccharide (LPS) to enter systemic circulation and trigger chronic low-grade inflammation (Lu et al., 2026; Jiang and Shen, 2023). This systemic inflammatory state may further aggravate synovial inflammation and cartilage degeneration in KOA. Moreover, gut microbiota-derived metabolites, particularly short-chain fatty acids (SCFAs) such as butyrate, have been shown to function as endogenous HDAC inhibitors. Butyrate can suppress HDAC3 activity and subsequently alleviate inflammatory responses while restoring tissue homeostasis (Parada-Venegas et al., 2025; Wilson et al., 2006). Therefore, gut microbiota dysbiosis-associated reduction in SCFA production may contribute to HDAC3 overactivation and KOA progression.

Traditional Chinese medicine (TCM) has long been applied in the prevention and treatment of KOA and has demonstrated unique advantages in alleviating symptoms and improving joint function. According to TCM theory, KOA is associated with liver and kidney deficiency, qi-blood insufficiency, and invasion of dampness-cold/heat pathogens (Zeng et al., 2023). Renshenguben (RSGB), a classical TCM prescription, possesses functions of consolidating constitution, restoring, antioxidant and immunomodulatory abilities. The RSGB prescription is made of *Panax ginseng* (Ren Shen), *Rehmannia glutinosa* (dried Di Huang), *Rehmannia glutinosa* (prepared Di Huang), *Cornus officinalis* (Shan Zhu Yu), *Rhizoma Dioscoreae* (Shan Yao), *Rhizoma Alismatis* (Ze Xie), *Cortex Moutan* (Mu Dan Pi), *Poria cocos* (Fu Ling), *Ophiopogon japonicus* (Mai Dong), and *Asparagus cochinchinensis* (Tian Dong). Modern pharmacological studies have shown that active components in RSGB formula exhibit anti-inflammatory, antioxidant, immunomodulatory, and tissue-repairing properties (Zhang et al., 2023; Feng et al., 2022). Furthermore, TCM formulas may exert therapeutic effects by modulating gut microbiota composition, improving intestinal barrier function, and restoring immune homeostasis (Feng et al., 2022; Gu et al., 2021). In addition to pharmacological therapy, exercise intervention has become an essential non-pharmacological treatment for KOA. Personalized exercise prescriptions, including aerobic exercise, resistance training, and flexibility training, can improve muscle strength, enhance joint stability, and reduce pain and stiffness. Experimental evidence demonstrated that exercise intervention can suppress inflammatory responses and protect cartilage by regulating multiple molecular pathways (de Sire et al., 2021; Lund et al., 2008). These findings suggest that combined pharmacological and exercise interventions may exert enhanced therapeutic effects on KOA.

However, although accumulating evidence has demonstrated the individual therapeutic benefits of TCM and exercise intervention, studies exploring their enhanced combined effects in KOA remain scarce. Therefore, the present study aims to elucidate the therapeutic efficacy and potential mechanisms of RSGB combined with exercise intervention in KOA from the perspective of the gut-joint axis. In particular, this study will investigate how gut microbiota modulation, immune-inflammatory responses, and the HDAC3/PPAR-γ signaling pathway contribute to cartilage degeneration and regeneration. Collectively, these findings may provide novel experimental evidence and a theoretical foundation for the development of comprehensive and individualized treatment strategies for KOA.

## Methods and materials

### Animal experiments

Forty-five six-week-old male specific pathogen-free (SPF) Sprague–Dawley (SD) rats weighing 180–220 g were purchased from the Experimental Animal Center of Southwest Medical University. All animal experimental protocols were reviewed and approved by the Animal Ethics Committee of Southwest Medical University, and all procedures were conducted in accordance with institutional guidelines for the care and use of laboratory animals. Housing conditions were 22 °C, 12 h light/dark cycle, 55 ± 10% humidity and free access to food and drinking water. After one week of acclimatization, the rats were randomly divided into five groups (*n* = 9 per group). On day 0, rats in group 1 (Control group) received an intra-articular injection of saline into the right knee, whereas rats in groups 2–5 received an intra-articular injection of 100 μL (2 mg) monosodium iodoacetate (MIA, Sigma, I2512) into the right knee to establish the KOA model (Saber et al., 2023). One week later, rats in groups 1 and 2 (MIA group) were orally administered saline for 4 consecutive weeks. During the same period, the 20-min swimming sessions were introduced to rats in groups 4 (Swimming group) and 5 (Swimming+RSGB group), while rats in groups 3 (RSGB group) and 5 received RSGB (Lunan Hope Pharmaceutical Co., Ltd.; National Drug Approval Number: Z10940013; National Drug Standards of the China National Medical Products Administration: WS3-260(Z-050)-2004(Z)-2021; Supplementary Drug Application Approval Notice No. 2022803129) by oral gavage at a dose of 8 mL/kg for 4 weeks. The preparation process of RSGB oral liquid was performed as follows. The volatile components of *Cortex Moutan* were extracted by steam distillation, and the distillate and residual herbal materials were retained for subsequent processing. *Panax ginseng* and *Cornus officinalis* were extracted twice by refluxing with 60% ethanol. The extracts were combined and filtered, and both the filtrates and residues were collected for further use. The residues of these two herbs, together with the remaining herbal components, were decocted with water twice. The aqueous extracts were filtered, pooled, and concentrated. Ethanol was then added to achieve a final concentration of 60%, and the resulting extract was combined with the ethanol extracts of *Panax ginseng* and *Cornus officinalis*, followed by filtration. Ethanol was removed by concentration, after which water was added, followed by boiling and subsequent filtration. Finally, the volatile components of *Cortex Moutan* and 100 g of syrup were added, the solution was adjusted to pH 7.0, and water was added to a final volume of 1,000 mL. The RSGB oral liquid was obtained after sterilization, with an equivalent crude herbal drug content of 0.483 g/mL (Feng et al., 2022). The rats’ weights were recorded weekly throughout the experimental period. After 24 h of the final intervention, knee joint swelling, von Frey and accelerated rotarod tests were performed to evaluate joint swelling severity, pain sensitivity and motor performance. All behavioral assessments were conducted at a consistent time of day to minimize the influence of circadian variation. Before testing, rats were allowed to acclimatize to the experimental environment and apparatus to reduce stress-related effects. Behavioral evaluations were performed by two investigators who were blinded to the experimental group assignments to minimize potential subjective bias. Rats were then anesthetized with pentobarbital sodium (60 mg/kg, intraperitoneal injection), and blood samples, liver and kidney tissues were collected for subsequent analyses. Three right knee joints from each group were fixed in 4% paraformaldehyde for micro-computed tomography (micro-CT) and histopathological examination. The remaining right knee joints were carefully dissected, and articular cartilage was scraped from the joint surface and stored at −80 °C for subsequent analyses.

### Hematological and serum biochemical analysis

At the end of the experimental period, blood samples were collected from rats under anesthesia. Part of the whole blood was transferred into anticoagulant tubes for hematological analysis, while the remaining blood was centrifuged at 3,000 rpm for 15 min at 4 °C to obtain serum for biochemical assays and ELISA. Hematological parameters were analyzed using an automated hematology analyzer (Mindray Bio-Medical Electronics Co., Ltd., BC-2800Vet). Serum biochemical parameters were determined using a fully automatic biochemical analyzer (Mindray Bio-Medical Electronics Co., Ltd., BS-460).

### Swimming protocol

To evaluate the therapeutic effects of swimming on KOA, rats were subjected to a swimming intervention beginning one week after MIA injection. The rats were placed in a circular water tank (30 °C, 100 cm depth) and allowed to swim freely for an initial duration of 5 min. The swimming duration gradually increased each day until reaching a maximum of 20 min per session. Rats underwent the swimming protocol 5 days per week for 4 consecutive weeks, while escape behavior was minimized throughout the training period (Saber et al., 2023; Lunz et al., 2008).

### Knee joint swelling assessment

Changes in knee joint diameter were determined using a digital vernier caliper by measuring both the right (MIA-injected) and left (uninjected) knee joints, and calculating the difference between the two measurements (Adães et al., 2014). For the Control group, the absolute value of the bilateral inter-limb difference was used due to the absence of a clearly defined affected side, to ensure consistent comparison among groups.

### Von Frey test for mechanical pain sensitivity

Before the initiation of behavioral testing, animals were transferred to the testing room and allowed to acclimate undisturbed for 1 h. Rats were then placed on a wide-gauge metal mesh platform and calibrated von Frey filaments were applied vertically from beneath the mesh to the plantar surface of the hind paw. The force gradually increased from lower to higher intensities until the rat exhibited a withdrawal response by rapidly lifting or flicking the paw away from the stimulus. The corresponding force (g) was recorded as the mechanical pain threshold. Each rat was tested at least three times with a 5 min interval between measurements, and the average value was calculated as the final result (Chaplan et al., 1994).

### Accelerated rotarod to evaluate motor performance

Motor performance was evaluated using an accelerating rotarod apparatus (Shenzhen Huayang Biotechnology Co., Ltd., HR420) according to the method described by Vonsy et al. with slight modifications (Vonsy et al., 2012). Before testing, animals were transferred to the behavioral testing room and allowed to acclimate undisturbed for 30 min. To familiarize the rats with the apparatus, training sessions were conducted 2 days before the formal experiment. After setting the experimental parameters, rats were placed on the stationary rotarod, and the device was started at a constant speed of 4 r/min to train the animals to maintain balance on the rotating rod. Each training session lasted 1–2 min and was repeated 2–3 times. During the formal test, rats were placed on the rotarod after all parameters had been set. The apparatus was initially operated at a constant speed, and once the animals maintained stable movement, the rotation speed gradually accelerated from 4 r/min over a period of 120 s. Running distance was recorded for each rat. Each animal was tested three times, and the average value was used for statistical analysis.

### Micro-CT scanning

Knee joints fixed in 4% paraformaldehyde were scanned using a micro-CT scanner (SkyScan 1276, Bruker). Three-dimensional reconstruction was subsequently performed using the manufacturer’s reconstruction software. For quantitative evaluation of bone microarchitecture, the following three-dimensional morphometric parameters were analyzed using the instrument’s analysis software: bone mineral density (BMD), bone volume fraction (BV/TV), trabecular thickness (Tb.Th), trabecular separation (Tb.Sp), and structural model index (SMI).

### ELISA

Serum C-reactive protein (CRP, Shanghai Zhuocai Biotechnology Co., Ltd., ZC-36085W), cartilage oligomeric matrix protein (COMP, Shanghai Zhuocai Biotechnology Co., Ltd., ZC-37149W), MMP-1 (Shanghai Zhuocai Biotechnology Co., Ltd., ZC-36745W), and MMP-13 (Shanghai Zhuocai Biotechnology Co., Ltd., ZC-36747W) levels were measured at one week after MIA injection and at the end of the experiment. In addition, serum levels of IL-1β (Shanghai Zhuocai Biotechnology Co., Ltd., ZC-36391W), NF-κB (Shanghai Zhuocai Biotechnology Co., Ltd., ZC-36696W), TNF-α (Shanghai Zhuocai Biotechnology Co., Ltd., ZC-37624W), interleukin-10 (IL-10, Shanghai Zhuocai Biotechnology Co., Ltd., ZC-36379W), diamine oxidase (DAO, Shanghai Zhuocai Biotechnology Co., Ltd., ZC-36566W), and LPS (Shanghai Zhuocai Biotechnology Co., Ltd., ZC-37600W) were measured at the end of the experiment.

### Real-time qPCR

Total RNA was isolated from tissue with Total RNA Extraction Kit (Biosharp, BL1365A) and reverse transcribed with reverse transcription reagent (Takara Biotechnology, Japan) according to the manufacturer’s protocol. Complementary DNA was used for real-time PCR with SYBR Premix Taq (Vazyme Biotech, China) in a Light Cycler (Roche Molecular Biochemicals, Switzerland). Three biological replicates were analyzed for each experimental group, and each biological sample was measured in technical triplicates. The average Ct value of the three technical replicates was used for subsequent analysis. Relative quantification of gene expression was performed with the comparative threshold method. Changes in mRNA expression levels were calculated after normalization to values for the β-actin calibrator gene. Gene expression values were represented using the 2-ΔΔCt method. The specificity of the amplification was confirmed by melting curve analysis. The primers used are shown below. HDAC3 (Forward: AGACCAGATCCGCCAGACCATC; Reverse: TCAGGACCTCTCTCTTCAGCATCG), PPAR-γ (Forward: CCATCGAGGACATCCAAGACAACC; Reverse: GTGCTCTGTGACAATCTGCCTGAG), β-actin (Forward: GGGAAATCGTGCGTGACATT; Reverse: GCGGCAGTGGCCATCTC).

### Western blotting analysis

Cartilage tissues were homogenized in lysis buffer containing 25 mM Tris–HCl (pH 7.6), 150 mM NaCl, 1 mM EDTA, 1% Triton X-100, 10% glycerol, and 1% protease inhibitor cocktail. Protein samples were separated by sodium dodecyl sulfate-polyacrylamide gel electrophoresis and subsequently transferred onto membranes (EpiZyme, China) using a wet transfer system. The membranes were incubated with primary antibodies overnight at 4 °C with gentle agitation. After incubation with the appropriate secondary antibodies, protein bands were visualized using an enhanced chemiluminescence detection kit (X-blot, China). The following primary antibodies were used: HDAC3 (1:2000, A2139SP, ABclonal), PPAR-γ (1:1000, A11183, ABclonal), β-actin (1:10000, LF208, EpiZyme). For quantification, cartilage samples from three independent animals per group were used as biological replicates (*n* = 3). Each sample was independently processed for protein extraction. Protein expression levels were quantified by densitometric analysis using ImageJ software and normalized to the corresponding loading control (β-actin).

### Histological and immunohistochemical analysis

Liver and kidney tissue samples were fixed in 4% paraformaldehyde for 48 h, followed by paraffin embedding, and 4-μm sections were prepared. Hematoxylin and eosin (H&E) staining was subsequently performed according to standard protocols, and histopathological changes were observed under a light microscope. Knee joints samples were fixed in 4% paraformaldehyde for 48 h. Specimens were decalcified in 20% EDTA (pH 7.4), followed by paraffin embedding, and 4-μm sections were prepared. H&E staining and Safranin O and Fast Green (SO&FG) staining were performed according to standard protocols. Knee sections were deparaffinized and subjected to antigen retrieval. The slides were then incubated overnight at 4 °C with primary antibodies against Collagen II (Col II, 1:1000, HA722733, HUABIO). The corresponding secondary antibody was applied to the slides and incubated for 2 h. Subsequently, 3,3′-diaminobenzidine (DAB) was used for chromogenic detection, followed by counterstaining with hematoxylin. Sections were randomly coded and evaluated independently by two blinded observers who were unaware of the experimental group assignments.

### 16S rRNA gene sequencing

Colon tissues and fecal samples from the colonic contents of rats in each group were collected into sterile EP tubes and stored at −80 °C. Genomic DNA was extracted according to the manufacturer’s instructions provided with the DNA extraction kit, and DNA quality was assessed by 1% agarose gel electrophoresis. The V3-V4 hypervariable regions of bacterial 16S rDNA were amplified by PCR using the following primers: 338F (5′-ACTCCTACGGGAGGCAGCAG-3′) and 806R (5′-GGACTACHVGGGTWTCTAAT-3′). The PCR product was extracted from 2% agarose gel and purified. Then quantified using Synergy HTX (Biotek, USA). Purified amplicons were pooled in equimolar amounts and paired-end sequenced on an Illumina NextSeq 2000 PE300 platform (Illumina, San Diego, USA) according to the standard protocols by Majorbio Bio-Pharm Technology Co. Ltd. (Shanghai, China). Bioinformatic analysis of the gut microbiota was carried out using the Majorbio Cloud platform.^1^ The *p* values from correlation analyses were corrected for multiple testing using the false discovery rate (FDR) method.

### Statistical analysis

GraphPad Prism 10.1.2 (La Jolla, USA) was used for analysis. One-way analysis of variance (ANOVA) was performed with recommended comparison test from at least three independent experiments. Tukey’s multiple comparisons test was performed after one-way ANOVA with correction for multiple comparisons. Statistical comparisons between two independent groups were performed using an independent samples *t*-test. *p* < 0.05 was considered statistically significant. Data is presented as mean ± SD.

## Results

### The safety of RSGB oral liquid

**Figure 1:**
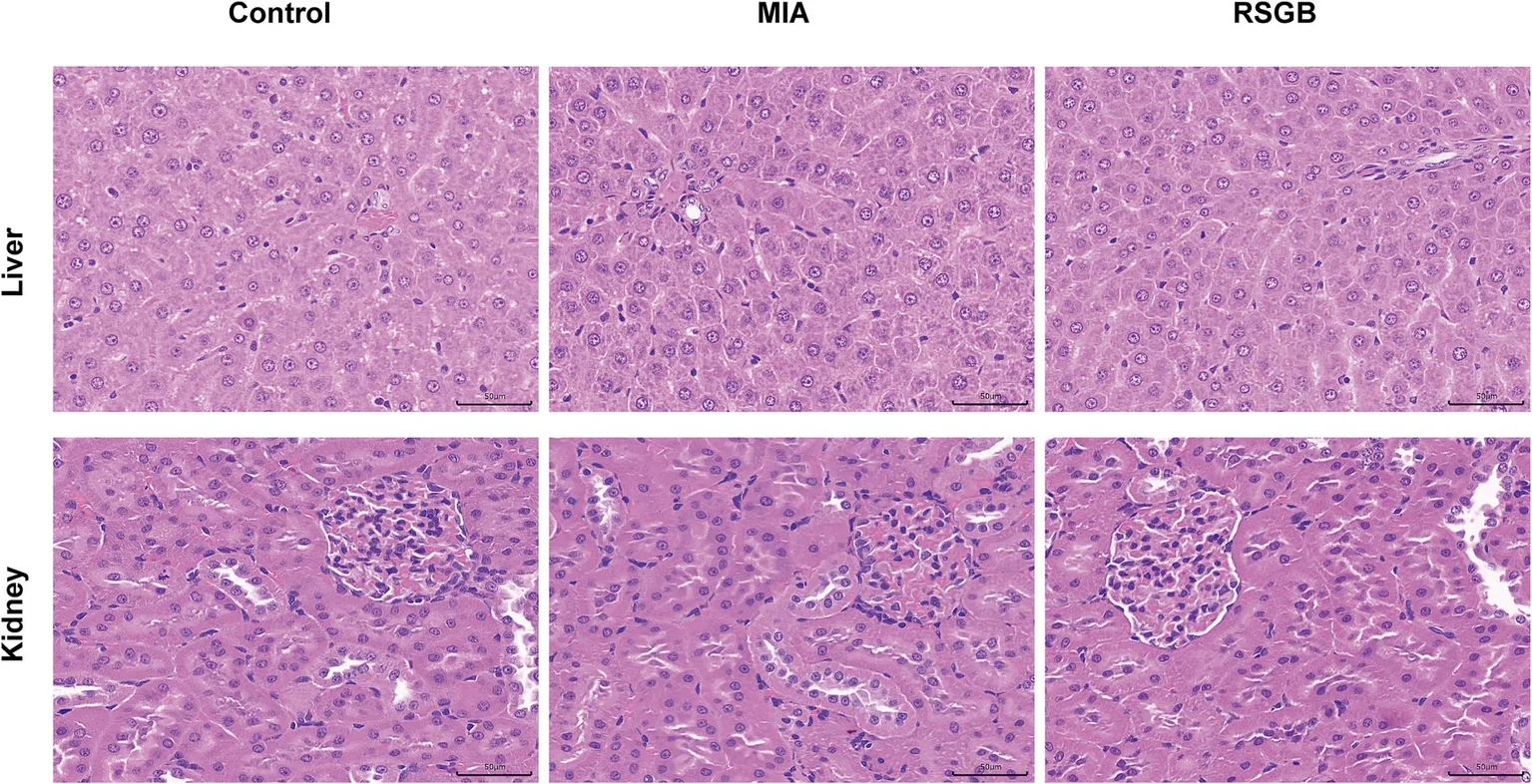
Histopathological analysis of liver and kidney tissues. Representative H&E-stained sections of liver and kidney from Control, MIA, and RSGB groups (*n* = 5). Liver tissues show intact capsules, normal hepatic cords, preserved central vein endothelium, and no obvious necrosis, congestion, or inflammatory infiltration. Kidney tissues display intact capsules, clear corticomedullary boundaries, normal glomerular and tubular structures, and no signs of degeneration, necrosis, fibrosis, or inflammatory infiltration. Scale bars: 50 μm.

The safety of 28 days of treatment of RSGB oral liquid (8 mL/kg) was investigated. Compared with the Control group, T-bil-V, Gran%, RBC, and HGB levels were slightly elevated in the MIA group, whereas all other hematological and biochemical parameters showed no obvious abnormalities. After drug treatment, no apparent abnormalities were observed in routine blood or biochemical examinations (Table 1). Besides, pathological analysis revealed no significant histopathological changes in major organs (Figure 1). These alterations may reflect inflammation-related responses rather than toxic effects, suggesting that the intervention exhibited favorable biosafety and did not induce significant systemic toxicity during the experimental period.

**Table 1:** Blood routine and biochemical indicators.

		Control	MIA	RSGB
Routine blood test				
WBC	10^9^/L	10.62 ± 0.48	11.04 ± 2.09	10.56 ± 2.09
LYMPH	10^9^/L	8.72 ± 0.30	7.52 ± 1.57	7.88 ± 2.3
LYMPH	%	82.04 ± 1.02	72.66 ± 1.34	68.38 ± 8.64
Mon	10^9^/L	0.28 ± 0.08	0.32 ± 0.08	0.32 ± 0.08
Mon	%	2.54 ± 0.21	3.12 ± 0.35	3.56 ± 0.69
Gran	10^9^/L	1.66 ± 0.18	2.46 ± 0.35	2.98 ± 0.43
Gran	%	15.44 ± 0.98	32.98 ± 5.71^****^	24.32 ± 1.10^**##^
RBC	10^12^/L	6.75 ± 0.34	8.47 ± 0.28^****^	7.91 ± 0.18^****#^
HGB	g/L	140.40 ± 3.21	159.40 ± 2.51^***^	150.80 ± 7.6^*#^
HCT	%	39.54 ± 0.68	45.24 ± 0.59	42.94 ± 1.64
MCV	fL	59.96 ± 2.33	54.12 ± 1.51	54.76 ± 2.11
MCH	pg	20.68 ± 1.42	18.92 ± 0.59	19.02 ± 1.13
MCHC	g/L	350.8 ± 6.83	348.2 ± 4.97	352.8 ± 7.33
RDW	%	9.96 ± 0.35	10.06 ± 0.31	9.74 ± 0.26
PLT	10^9^/L	1060.40 ± 25.84	976.80 ± 87.83	1006.40 ± 124.46
MPV	fL	5.08 ± 0.23	4.90 ± 0.25	5.08 ± 0.29
PDW		16.46 ± 0.15	16.10 ± 0.4	16.26 ± 0.11
PCT	%	0.54 ± 0.02	0.48 ± 0.07	0.49 ± 0.09
Biochemical test				
ALB	g/L	30.54 ± 0.61	30.78 ± 1.34	30.44 ± 0.45
ALT	U/L	56.78 ± 4.42	60.48 ± 1.03	55.06 ± 12.38
AST	U/L	123.12 ± 10.84	136.84 ± 11.87	136.60 ± 3.47
CREA	umol/L	23.44 ± 0.90	30.64 ± 1.47	29.82 ± 1.10
HDL-C	mmol/L	1.17 ± 0.23	1.06 ± 0.13	0.85 ± 0.08
LDH	U/L	1132.38 ± 53.82	1269.92 ± 259.85	1275.62 ± 239.85
LDL-C	mmol/L	0.34 ± 0.08	0.33 ± 0.02	0.29 ± 0.04
T-bil-V		0.14 ± 0.02	0.65 ± 0.26^***^	0.54 ± 0.07^**^
TC	mmol/L	1.80 ± 0.34	1.66 ± 0.14	1.43 ± 0.18
TG	mmol/L	1.34 ± 0.81	0.91 ± 0.52	0.49 ± 0.18
TP	g/L	53.66 ± 4.94	55.48 ± 3.19	53.76 ± 0.70
UREA	mmol/L	5.39 ± 0.90	6.07 ± 0.40	4.90 ± 0.25

Data are presented as mean ± SD (*n* = 5). *: vs Control *p* < 0.05; **: vs Control *p* < 0.01; ***: vs Control *p* < 0.001; ****: vs Control *p* < 0.0001; ^##^: vs MIA *p* < 0.01; ^#^: vs MIA *p* < 0.05. *p*-values were determined by one-way ANOVA.

### Combining RSGB and swimming reduced OA-related swelling and pain and improved articular motor performance

**Figure 2:**
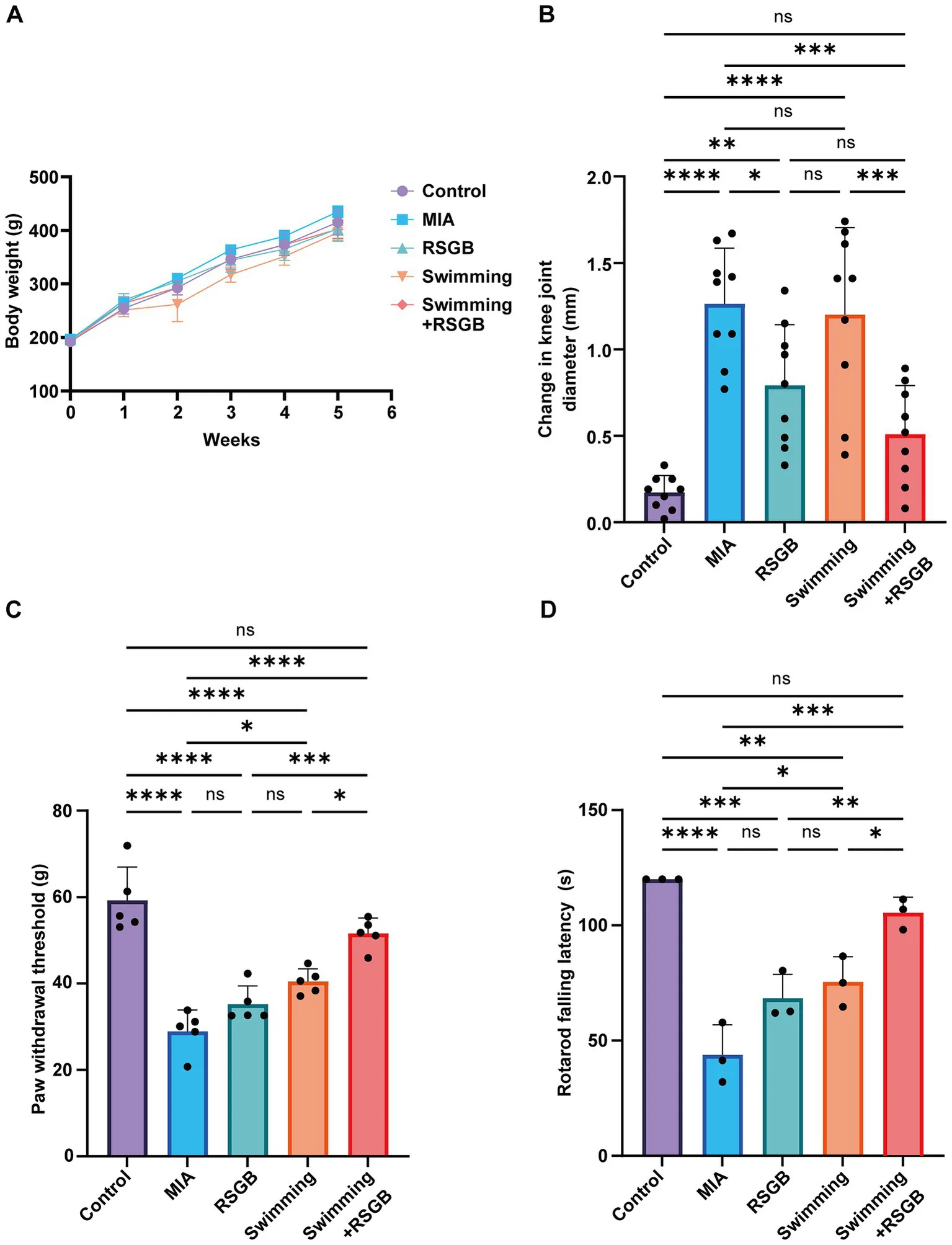
Analysis of body weight, change in knee joint diameter, Von Frey paw withdrawal threshold, and rotarod falling latency in rats subjected to 4 weeks of swimming, RSGB or swimming+RSGB. **(A)** Body weight changes in each group during the experimental period (*n* = 9). **(B)** Change in knee joint diameter in each group during the experimental period (*n* = 9). **(C)** Paw withdrawal thresholds in each group at the end of the experiment (*n* = 5). **(D)** Motor performance in each group (*n* = 3). *p < 0.05; **p < 0.01; ***p < 0.001; ****p < 0.0001; ns, not significant. *p*-values were determined by one-way ANOVA.

Body weight was measured weekly throughout the study to monitor the growth status of the rats (Figure 2A). The results indicated that the interventions did not affect the normal growth of the rats throughout the experimental period. The degree of knee joint swelling levels in each group were assessed following MIA induction and treatment. Both RSGB-treated groups showed a significant reduction in swelling, with the combined treatment group demonstrating the most pronounced improvement (Figure 2B). Compared with the Control group, MIA-treated rats exhibited a significantly lower response threshold to mechanical stimulation, indicating increased tactile hypersensitivity. Compared with the MIA group, Swimming+RSGB group significantly reduced tactile hypersensitivity. Although swimming or RSGB group also showed a trend toward increasing the paw withdrawal threshold, their effects were less pronounced than those observed in the Swimming+RSGB group (Figure 2C). Besides, compared with the Control group, MIA-treated rats exhibited significantly impaired motor performance, whereas all treatment groups showed varying degrees of recovery compared with the MIA group. Notably, the combined RSGB and swimming exercise group demonstrated superior motor performance (Figure 2D).

### Combining RSGB and swimming treatment suppressed inflammation and improved serum marker levels related to KOA

**Figure 3:**
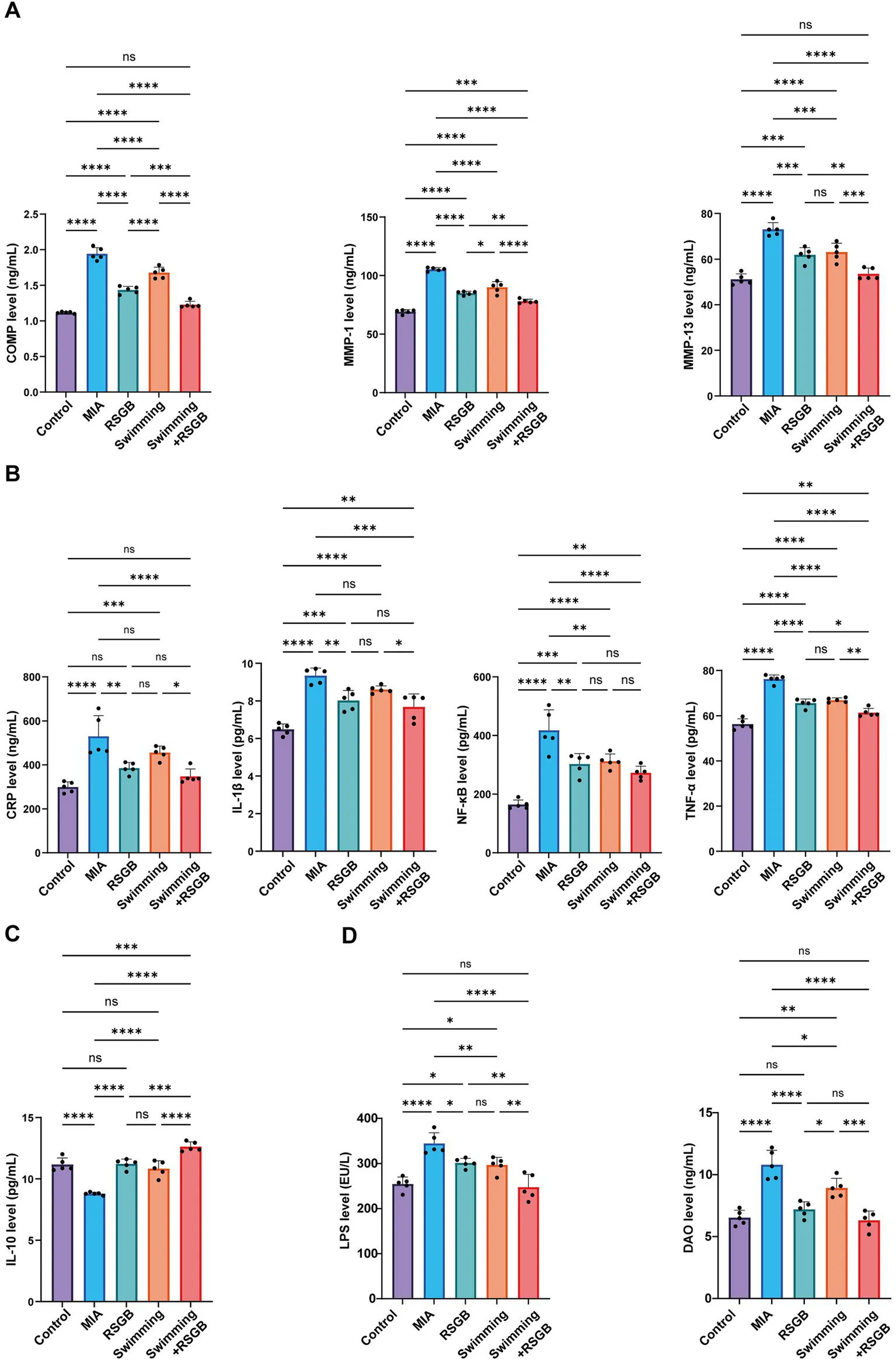
Levels of COMP, MMP-1, MMP-13, CRP, IL-1β, NF-κB, TNF-α, IL-10, LPS and DAO in the serum of the rats subjected to 4 weeks of swimming, RSGB or swimming+RSGB. **(A)** Serum biomarkers of cartilage and joint tissue damage in KOA (*n* = 5). **(B,C)** Serum pro-inflammatory and anti-inflammatory biomarkers (*n* = 5). **(D)** Serum biomarkers of intestinal permeability and gut barrier integrity.*p < 0.05; **p < 0.01; ***p < 0.001; ****p < 0.0001; ns, not significant. *p*-values were determined by one-way ANOVA.

Serum levels of COMP, CRP, MMP-1, MMP-13, IL-1β, IL-10, NF-κB, TNF-α, LPS, and DAO were assessed in all experimental groups (Figure 3). Compared with the Control group, MIA-induced KOA rats exhibited significantly elevated levels of COMP, CRP, MMP-1, MMP-13, IL-1β, NF-κB, TNF-α, LPS, and DAO, accompanied by a reduction in IL-10, indicating enhanced cartilage degradation, systemic inflammation, and impaired intestinal barrier integrity. Treatment with either RSGB or swimming alone partially reversed these alterations. Notably, the combined Swimming+RSGB intervention produced the most pronounced effects, characterized by the greatest reductions in pro-inflammatory cytokines, matrix-degrading enzymes, and gut permeability markers, together with a marked restoration of IL-10 levels. These findings suggest that the combined intervention not only attenuated inflammatory and catabolic responses associated with KOA but also ameliorated alterations in indicators associated with intestinal barrier dysfunction, thereby potentially reducing gut-derived inflammatory stimuli and contributing to the restoration of gut-joint homeostasis.

### Combining RSGB and swimming treatment reverses the dysregulated expression of PPAR-γ and HDAC3

**Figure 4:**
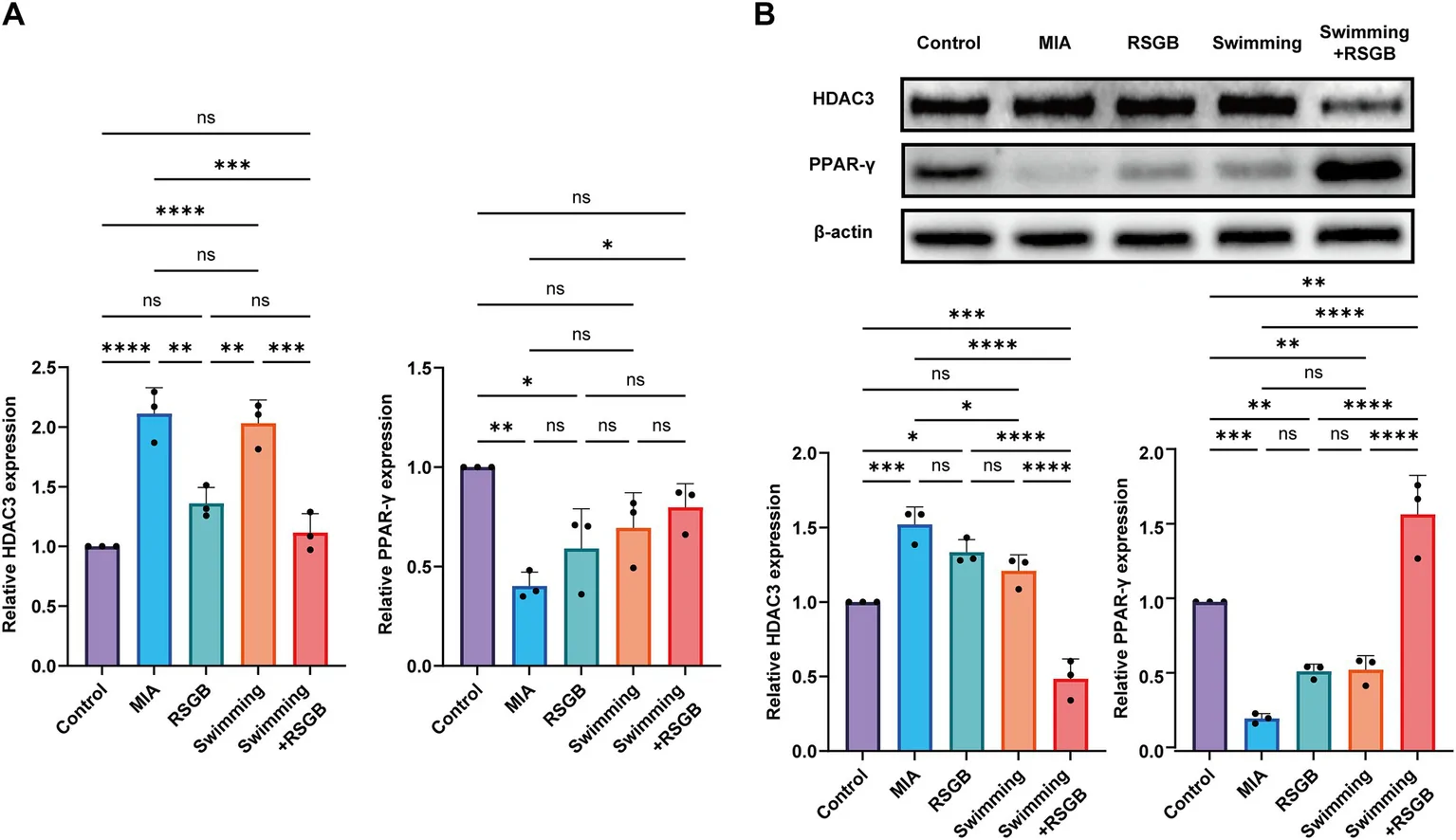
Expression levels of HDAC3 and PPAR-γ in the knee articular cartilage of rats after 4 weeks of swimming, RSGB or swimming+RSGB. **(A)** Relative mRNA expression levels of HDAC3 and PPAR-γ in the knee articular cartilage (*n* = 3). **(B)** Representative western blot images of HDAC3 and PPAR-γ proteins (*n* = 3). *p < 0.05; **p < 0.01; ***p < 0.001; ****p < 0.0001; not significant. *p*-values were determined by one-way ANOVA.

Following MIA-induced KOA, PPAR-γ expression was significantly decreased, whereas HDAC3 expression (detected as a band at approximately 55–70 kDa) was markedly increased compared with the Control group (Figure 4). All treatment groups partially ameliorated these alterations compared with the MIA group.

### Combining RSGB and swimming treatment improves bone microarchitecture and ameliorates histopathological damage in MIA-induced KOA rats

**Figure 5:**
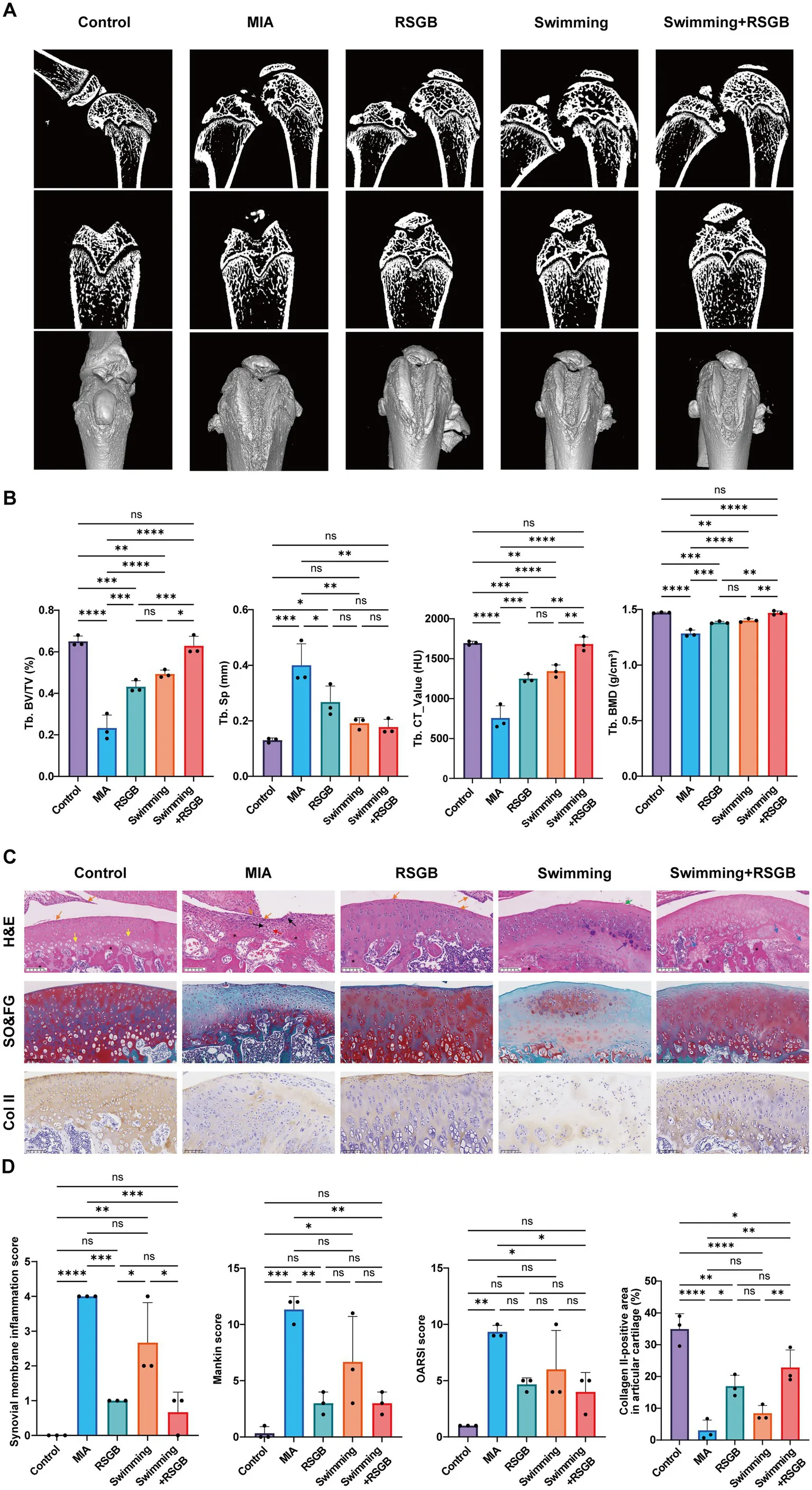
Micro-CT and histological analysis of knee joint in different treatment groups. **(A)** Representative micro-CT images and 3D reconstructed images of each group (*n* = 3). **(B)** Quantitative analysis of Tb.BV/TV, Tb.BMD, Tb.CT_value and Tb.Sp (*n* = 3). **(C)** Representative micrographs of H&E staining, SO&FG staining and immunohistochemical staining of Col II in articular cartilage. Scale bar = 100 μm. Orange arrows: Synovium. Yellow arrows: Tidemark. *: Bone or subchondral bone. Red arrows: Blood vessels. Black arrows: Inflammatory cells. Green arrows: Detached synovial cells. Purple arrows: Enhanced basophilia of chondrons. Blue arrows: Necrotic chondrocytes. **(D)** The synovial membrane inflammation, Mankin and Osteoarthritis Research Society International score for different groups. Semi-quantitative analysis of the Col II-positive area (%) (*n* = 3). *p < 0.05; **p < 0.01; ***p < 0.001; ****p < 0.0001; ns, not significant. *p*-values were determined by one-way ANOVA.

Micro-CT analysis revealed clear structural deterioration in the MIA group compared with the Control group. Tb.BV/TV, Tb.BMD, and Tb.CT_value were all significantly reduced following MIA injection, indicating a loss of subchondral bone mass and microarchitectural integrity. In contrast, Tb.Sp was significantly increased, suggesting enhanced trabecular separation and deterioration of bone microstructure. Following therapeutic intervention, rats treated with swimming or RSGB alone showed varying degrees of improvement compared with the MIA group. Notably, the Swimming+RSGB group demonstrated the most pronounced protective effect, showing the greatest restoration of Tb.BV/TV, Tb.BMD, and Tb.CT_value, indicating superior preservation of subchondral bone microstructure among all treatment groups (Figures 5A,B). H&E staining revealed marked pathological changes in the knee joints of MIA-induced KOA rats. The Control group maintained a completely normal microscopic structure of the knee joint. The synovium was thin and uniform without any signs of hyperplasia or inflammatory cell infiltration. The cartilage morphology was intact, the chondrocytes and matrix appeared normal, and the tidemark was clearly distinguishable. In contrast, the MIA group exhibited pronounced synovial hyperplasia accompanied by extensive inflammatory cell infiltration. The cartilage surface was highly irregular, with severe structural disruption, disorganization, and matrix loss. Localized full-thickness cartilage damage was observed, leaving only subchondral bone beneath the hyperplastic synovial tissue, along with visible neovascularization. Following different interventions, the histopathological features of KOA rats were ameliorated to varying degrees. Consistent with these microscopic observations, synovitis, Mankin, and OARSI scores were significantly increased in the MIA group compared with the Control group. While individual interventions partially alleviated these histopathological alterations, the combined treatment further improved pathological outcomes, as evidenced by significantly reduced synovitis, Mankin, and OARSI scores compared with the MIA group. Immunohistochemical analysis demonstrated a significant reduction in Col II expression in the MIA group relative to the Control group. In contrast, RSGB and Swimming+RSGB treatments all significantly increased Col II-positive staining compared with the MIA group. The combined intervention showed a trend toward higher Col II expression (Figures 5C,D). These results indicate that both RSGB and swimming exert protective effects against cartilage matrix degradation in KOA.

### Combining RSGB and swimming treatment modulates KOA-associated gut dysbiosis and promotes a healthier microbial profile

Five fecal samples collected from the colons of rats in each group were randomly selected for 16S rRNA gene sequencing. After quality filtering and optimization, a total of 1,601,724 high-quality sequences were obtained, comprising 738,060,710 bases with an average sequence length of 460.68 bp. Taxonomic annotation and clustering analysis identified 12 phyla, 19 classes, 45 orders, 81 families, 193 genera, 338 species, and 12,596 amplicon sequence variants (ASVs).

**Figure 6:**
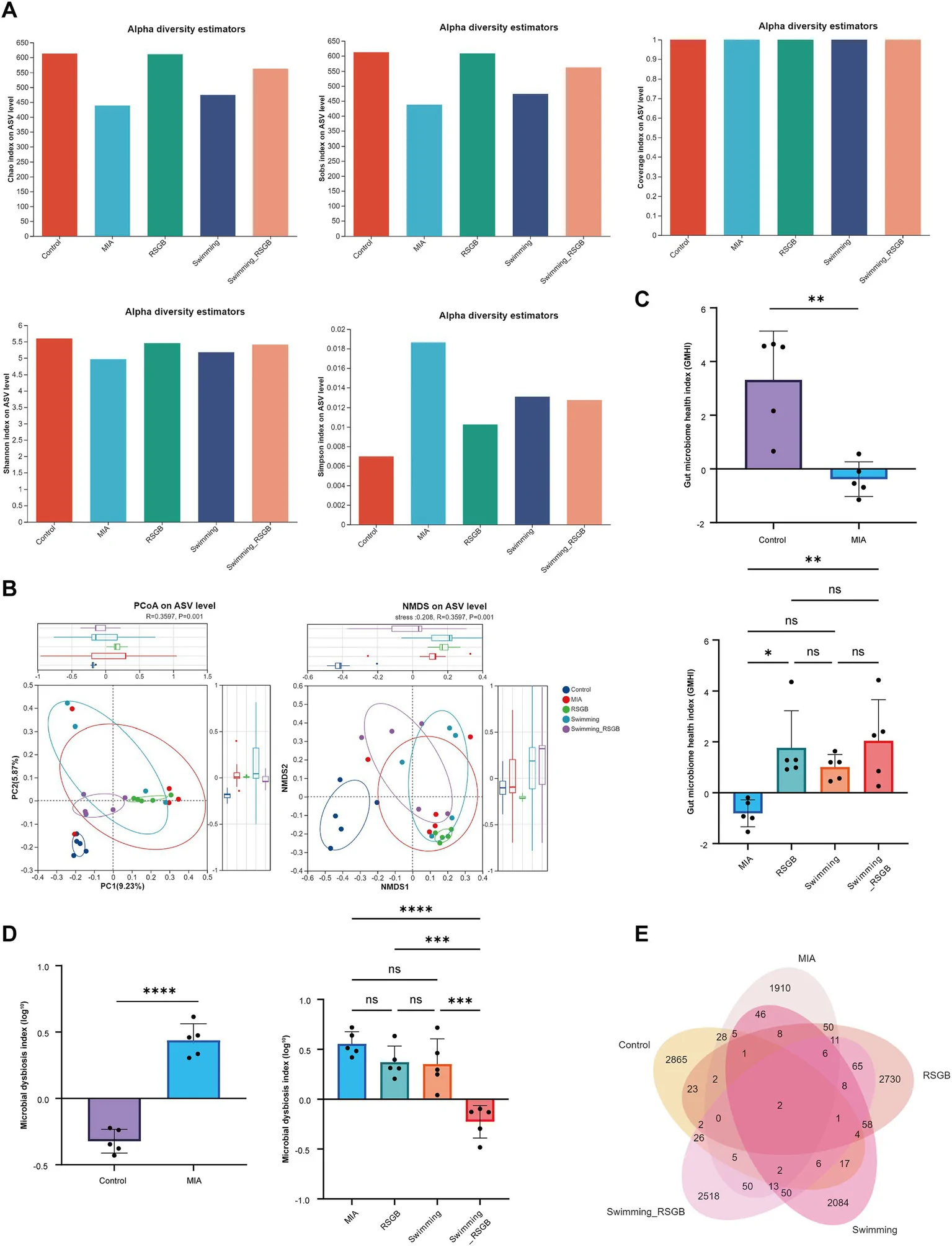
Comprehensive analysis of the effects of different interventions on the structure and function of the gut microbiota in rats. **(A)** Alpha diversity analysis. **(B)** Beta diversity analysis. **(C)** Comparison of GMHI among different groups. **(D)** Comparison of MDI among different groups. **(E)** Venn diagram showing the distribution of ASVs among different groups. *p < 0.05; **p < 0.01; ***p < 0.001; ****p < 0.0001; ns, not significant. *p*-values were determined by one-way ANOVA or independent samples *t*-test.

The Chao index reflected species richness within a sample, with higher values indicating a greater number of species. The Chao index was close to the Sobs index, suggesting sufficient sequencing depth and indicating that nearly all species present in the sample had been captured. The Coverage index was used to assess sequencing completeness, and values approaching 1 indicated that the majority of microbial taxa present in the sample had been successfully captured. The Shannon index evaluated both species richness and evenness. Compared with the Control group, the MIA group exhibited reduced gut microbial diversity. In contrast, both RSGB-treated groups showed recovery of microbial diversity and exercise-only group also demonstrated improvement. The Simpson index placed greater emphasis on the evenness of dominant species. The highest Simpson index observed in the MIA group suggested that the microbial community structure was characterized by the predominance of a few dominant taxa, reflecting reduced microbial diversity. All treatment groups showed improvement in this parameter, indicating partial restoration of microbial community structure (Figure 6A).

β diversity analysis was performed to further assess differences in microbial community composition among groups. Principal coordinates analysis (PCoA) and non-metric multidimensional scaling (NMDS) were used to visualize the relationships among samples (Figure 6B). Shorter distances between sample points indicated greater similarity in microbial community composition, whereas larger distances reflected greater compositional differences. Samples from the Control group clustered tightly and were distinctly separated from the other groups, indicating a stable microbial community structure with low inter-individual variation. In contrast, samples from the MIA group were more widely dispersed, suggesting pronounced microbial dysbiosis, greater inter-sample variability, and substantial disruption of community structure. The RSGB group exhibited relatively tight clustering and was clearly separated from both the Control and MIA groups, indicating that RSGB intervention markedly altered the gut microbial community and promoted a more stable microbial structure. The Swimming group showed moderate clustering and occupied a distinct position from both the Control and MIA groups, suggesting that exercise also reshaped the gut microbiota, although the pattern of change differed from that induced by RSGB treatment. The RSGB+Swimming group was distributed in an intermediate region and partially overlapped with both the RSGB and Swimming groups. These findings suggest that the combined intervention reconstructed the gut microbial community toward a healthier state while inducing a distinct pattern of microbial diversity changes. The results of NMDS analysis were largely consistent with those of PCoA, showing a similar clustering pattern among the groups.

The gut microbial health index (GMHI) reflects the overall health status of the gut microbiota, with higher values indicating a microbial community composition closer to that of healthy controls (Figure 6C). The microbial dysbiosis index (MDI) reflects the degree of microbial imbalance, with higher values indicating more severe dysbiosis (Figure 6D). Both sets of results indicated that, compared with the Control group, the MIA group exhibited a marked disruption of the gut microbiota structure and a significant increase in microbial dysbiosis. In contrast, the combined intervention group significantly improved microbiota imbalance and showed a restoration trend closer to the normal state. The Venn diagram revealed the shared and unique ASVs among the five groups, providing an overview of the similarities and differences in microbial community composition (Figure 6E). Only 2 ASVs were common to all groups, whereas 2,865, 1910, 2,730, 2084, and 2,518 unique ASVs were detected in the Control, MIA, RSGB, Swimming, and RSGB+Swimming groups.

**Figure 7:**
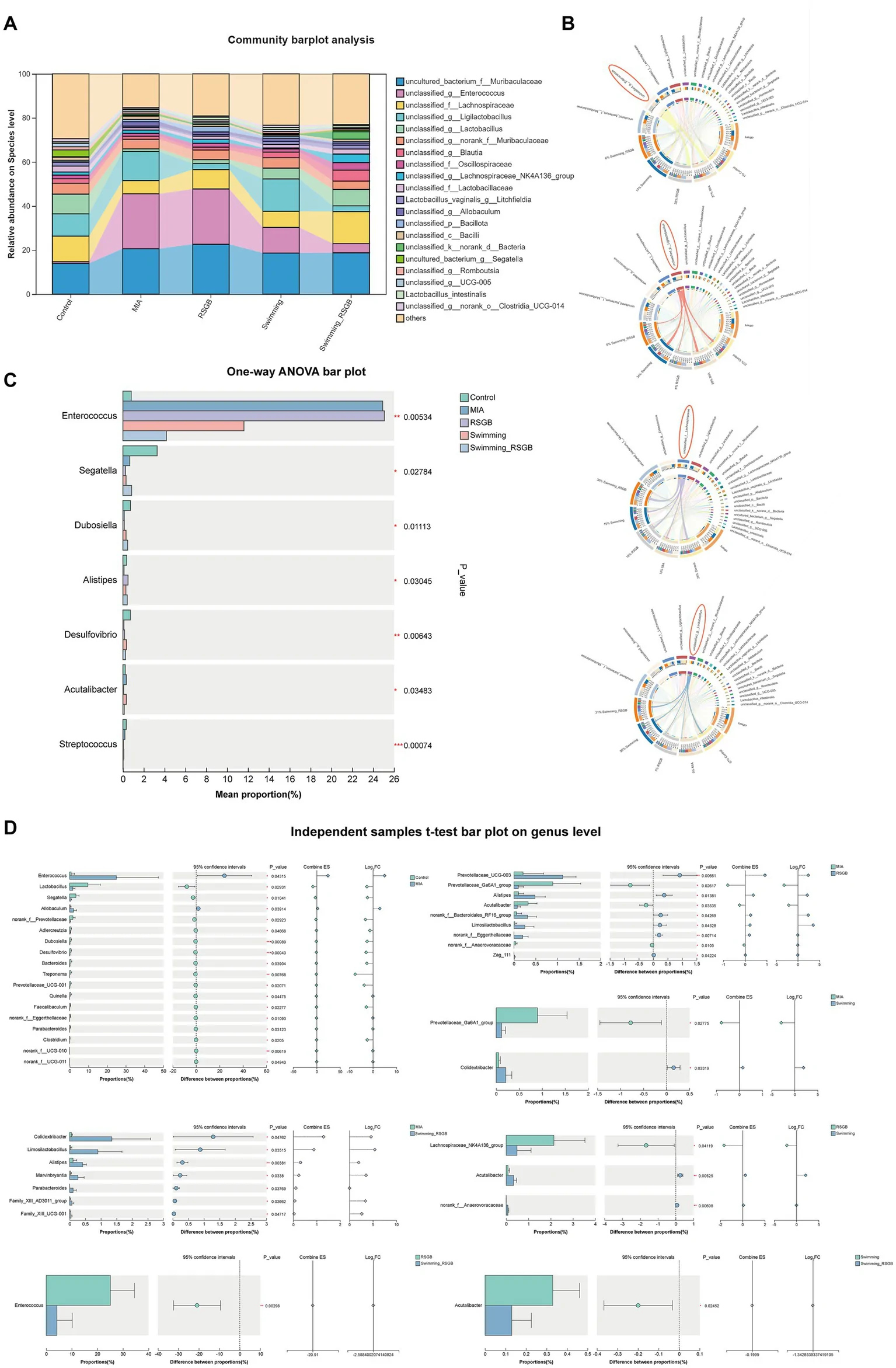
Analysis of gut microbiota composition and species differences among groups. **(A,B)** Gut microbial community composition shown by bar plot and circos plot. **(C,D)** Comparative analysis of species differences among groups. *p < 0.05; **p < 0.01; ***p < 0.001; ****p < 0.0001; ns, not significant. *p*-values were determined by one-way ANOVA or independent samples *t*-test.

At the species level, compositional analysis of the gut microbiota revealed differences among groups following different interventions (Figures 7A,B). Compared with the Control group, the MIA group showed a trend toward increased abundance of *Enterococcus*, while Swimming and Swimming+RSGB groups appeared to reduce their relative abundance. In contrast, *Lachnospiraceae* exhibited a decreasing trend in the MIA group compared with the Control group, whereas all intervention groups showed a tendency toward recovery. Similarly, *Ligilactobacillus* appeared to be enriched in the MIA group and showed a downward trend following RSGB and Swimming+RSGB interventions. Conversely, *Lactobacillus* showed a decreasing trend in the MIA group, while all treatment groups displayed an apparent increase. Overall, KOA induction induced gut microbiota dysbiosis characterized by an apparent shift toward increased potentially pathogenic bacteria (e.g., *Enterococcus*) and decreased beneficial SCFA-producing bacteria (e.g., *Lachnospiraceae* and *Lactobacillus*). Interventions appeared to partially reverse this dysbiosis, suggesting a potential restoration of microbial homeostasis, with the combined treatment group showing the most pronounced recovery trend. At the genus level, differential abundance analysis among groups further revealed distinct alterations in gut microbial composition (Figures 7C,D). Compared with the Control group, the MIA group exhibited distinct shifts in multiple taxa, characterized by an increased abundance of potential pathogenic bacteria and a decreased abundance of beneficial SCFA-producing bacteria. Following different interventions, these microbial alterations were partially reversed. In particular, the relative abundance of beneficial taxa was increased, whereas that of potentially pathogenic bacteria was reduced compared with the MIA group. Notably, the combined intervention group showed a more pronounced restoration of gut microbiota composition, with a trend toward normalization of microbial structure. Overall, these findings indicate that intervention treatment can modulate KOA-associated gut dysbiosis, promoting a shift toward a healthier microbial profile.

**Figure 8:**
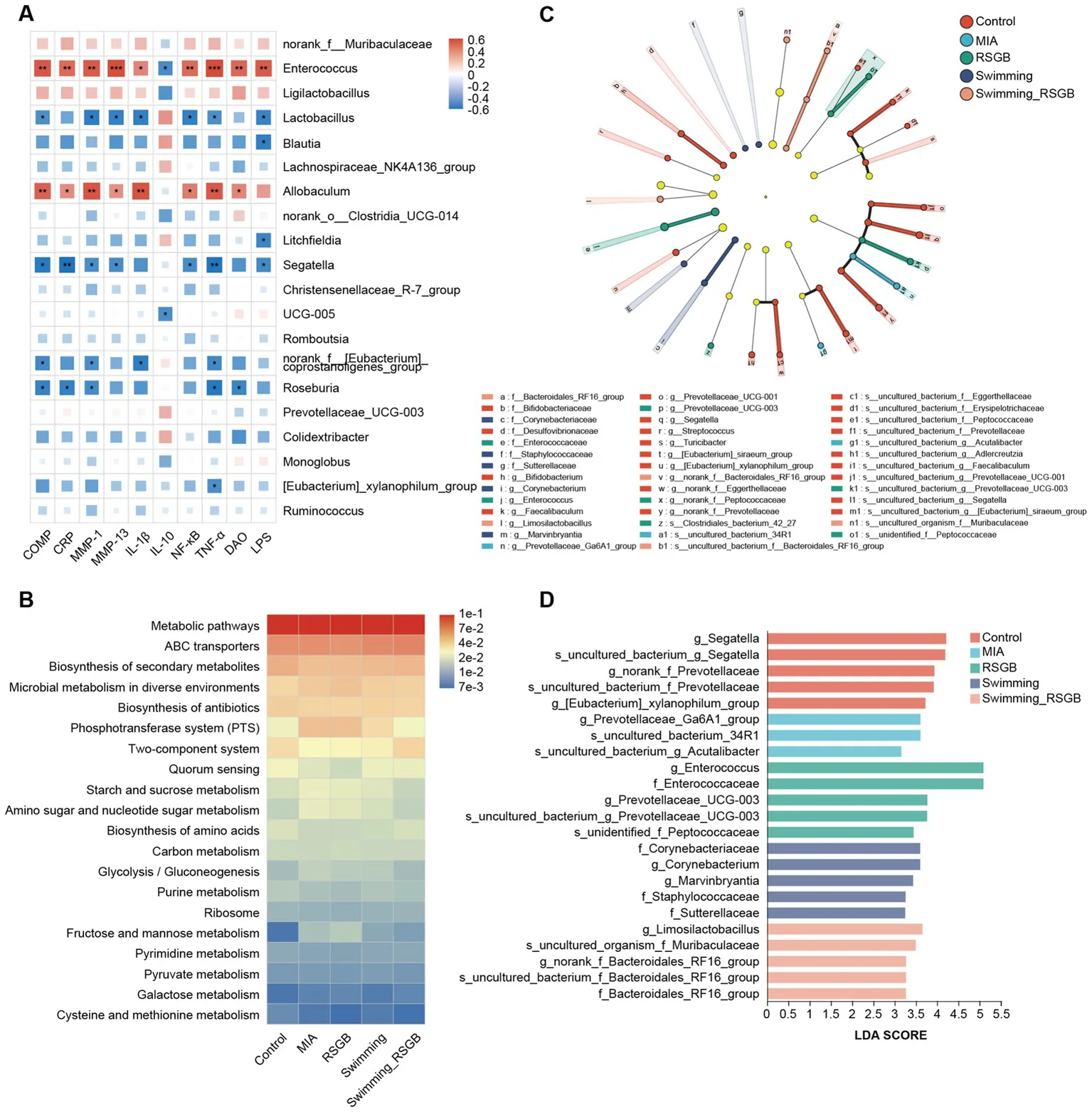
Correlation analysis, functional prediction, and biomarker identification of gut microbiota alterations in KOA rats following different interventions. Spearman correlation heatmap showing the associations between the relative abundance of dominant bacterial genera and inflammation-related and intestinal barrier-associated indicators **(A)**. Color gradients indicate the strength and direction of correlations, with red representing positive correlations and blue representing negative correlations. Tax4Fun-based prediction of microbial functional profiles based on KEGG level 3 pathways **(B)**. LEfSe analysis identified differentially abundant microbial taxa from the family to species levels among all experimental groups **(C,D)**. The cladogram illustrates the phylogenetic distribution of discriminative taxa enriched in each group, while the LDA score plot displays the taxa with significant discriminatory power among groups (LDA score > 3).

To further elucidate the potential link between KOA-induced gut microbiota dysbiosis and inflammatory responses, genus-level microbial abundance was correlated with inflammation-related and intestinal barrier-associated indicators (Figure 8A). The heatmap revealed multiple significant correlations between specific microbial taxa and inflammatory factors, suggesting a potential interaction between gut microbial alterations and host inflammatory status during KOA progression. Among the identified microbial genera, Enterococcus and Allobaculum exhibited distinct correlation patterns with inflammation-related indicators and was considered a potential inflammation-associated taxon. In contrast, beneficial or homeostasis-associated genera, including Lactobacillus and Roseburia, showed negative associations with inflammatory markers, suggesting their potential involvement in maintaining intestinal microbial balance and regulating inflammatory responses. Additionally, Segatella, a metabolically active member of the gut microbiota, also displayed correlations with inflammation-related parameters, indicating its possible contribution to KOA-associated microbial and immune alterations. These findings suggest that gut microbiota dysregulation may be closely linked to systemic inflammatory regulation during KOA development. Intervention-induced restoration of beneficial microbial communities may contribute to the attenuation of inflammatory responses and improvement of joint pathological conditions. KEGG level 3 pathways associated with microbial metabolism, including microbial metabolism in diverse environments, carbon metabolism, biosynthesis of amino acids, and biosynthesis of secondary metabolites, exhibited differential distribution patterns among groups (Figure 8B). In addition, pathways involved in carbohydrate metabolism, such as starch and sucrose metabolism, fructose and mannose metabolism, galactose metabolism, and glycolysis/gluconeogenesis, were altered following KOA induction and intervention. These metabolic pathways are closely related to microbial carbohydrate utilization and may influence the metabolic potential of SCFA-associated bacterial communities (Morrison and Preston, 2016; Kingkaw et al., 2022). In particular, the altered abundance of SCFA-associated genera, including Roseburia, Allobaculum, and Lactobacillus, together with changes in carbohydrate metabolism-related pathways, suggests that KOA progression may be accompanied by alterations in the predicted capacity of gut microbiota to participate in SCFA-related metabolic processes. The predicted functional alterations were also accompanied by changes in microbial adaptation and interaction pathways, including ABC transporters, phosphotransferase system (PTS), two-component system, and quorum sensing, suggesting that KOA-associated dysbiosis may influence microbial ecological functions beyond taxonomic composition. Compared with the MIA group, RSGB treatment, swimming exercise, and combined intervention partially shifted the predicted microbial functional profile toward that observed in the Control group, indicating potential restoration of microbial metabolic homeostasis and SCFA-associated functional capacity. LEfSe analysis was subsequently performed to identify discriminatory microbial taxa contributing to the differences among experimental groups (Figures 8C,D). The cladogram revealed distinct phylogenetic distributions of differential taxa among the five groups, indicating substantial remodeling of gut microbial composition following KOA induction and intervention treatments. At different taxonomic levels, several bacterial lineages exhibited significant group-specific enrichment patterns. The LEfSe linear discriminant analysis (LDA) score analysis further identified taxa with the greatest discriminatory power among groups. Several microbial features, including Enterococcus, Enterococcaceae, Bacteroidales_RF16_group, Limosilactobacillus, Sutterellaceae, exhibited strong contributions to group separation, indicating their potential value as microbial signatures associated with KOA status and treatment responses. These findings further confirmed that KOA induction and subsequent interventions resulted in characteristic shifts in gut microbial composition.

## Discussion

KOA is a multifactorial chronic degenerative joint disease driven by mechanical wear, systemic low-grade inflammation, metabolic abnormalities, and immune dysregulation (Xu et al., 2024). The present study aimed to evaluate the therapeutic efficacy and underlying molecular mechanisms of RSGB combined with swimming in a MIA-induced KOA rat model. Our findings demonstrated that the combination of RSGB and swimming further alleviated joint swelling, reduced mechanical pain sensitivity, and improved motor performance more effectively than either intervention alone. Furthermore, the combined treatment was more effective in protecting subchondral bone microarchitecture, suppressed systemic inflammation, and restored gut microbiota homeostasis. The progression of KOA is deeply associated with elevated pro-inflammatory cytokines and catabolic enzymes that degrade the cartilage extracellular matrix (Li and Wu, 2021). In our study, MIA induction triggered a robust inflammatory response, marked by elevated serum levels of TNF-α, IL-1β, and NF-κB, which subsequently upregulated the expression of matrix-degrading enzymes, including MMP-1, MMP-13, and COMP. Treatment with RSGB and swimming effectively reversed these biochemical alterations and increased the anti-inflammatory cytokine IL-10. The structural benefits of this anti-inflammatory effect were confirmed by micro-CT, histopathological and immunohistochemical analyses.

In addition to systemic inflammation, accumulating evidence suggests that impairment of the intestinal barrier contributes to the pathogenesis of KOA through the gut-joint axis. DAO and LPS are widely recognized biomarkers of intestinal permeability and barrier dysfunction (Liu et al., 2020; Gonzalez-Sarrias et al., 2018). In the present study, MIA-induced KOA significantly increased serum DAO and LPS levels, which are indirect indicators associated with intestinal barrier dysfunction and enhanced translocation of gut-derived endotoxins into the circulation. Such alterations may promote chronic low-grade systemic inflammation and accelerate joint degeneration. Notably, both RSGB and swimming reduced serum DAO and LPS levels, whereas the combined intervention produced the most pronounced effect. These findings suggest that RSGB combined with swimming effectively reduces serum markers associated with intestinal barrier dysfunction, limits the leakage of bacterial products into the bloodstream, and attenuates gut-derived inflammatory stimulation. Crucially, our study provides novel insights into the “gut-joint axis” by establishing a mechanistic link between gut microbiota dysbiosis, HDAC3, and PPAR-γ in KOA pathogenesis. Emerging evidence indicates that gut microbiota-derived metabolites, specifically SCFAs like butyrate, act as endogenous inhibitors of HDAC3 (Parada-Venegas et al., 2025; Wilson et al., 2006). Our 16S rRNA sequencing revealed that KOA induction led to marked microbial dysbiosis, notably decreasing the abundance of beneficial SCFA-producing bacteria, such as *Lachnospiraceae* and *Lactobacillus*. This reduction in SCFA-producing bacteria may indicate altered microbial metabolic capacity, which could potentially influence HDAC3 regulation through changes in microbiota-derived metabolites. However, the specific metabolites involved require further investigation. Consequently, we observed a significant overactivation of HDAC3 in the osteoarthritic cartilage. Accumulating evidence confirms that the nuclear translocation of HDAC3 is essential for reducing the transcriptional activity of PPAR-γ, whereas inhibiting HDAC3 activity leads to protein acetylation, thereby activating PPAR-γ (Ding et al., 2020; Fajas et al., 2002; Gao et al., 2006). Consistent with this mechanism, we observed a significant overactivation of HDAC3 and a concurrent, marked downregulation of PPAR-γ in the osteoarthritic cartilage of our KOA model. This critical disruption of the HDAC3/PPAR-γ balance facilitated the unchecked release of MMPs and pro-inflammatory cytokines, ultimately accelerating joint degeneration. The combined intervention of RSGB and swimming effectively broke this pathological cycle. The treatments reshaped the gut microbial community toward a healthy state, restoring the populations of SCFA-producing bacteria (*Lachnospiraceae* and *Lactobacillus*) and suppressing potentially pathogenic taxa like *Enterococcus*. We propose that this microbiota restoration may enhance SCFA-related metabolic potential, which may contribute to the suppression of HDAC3 overactivation in the joint and the restoration of PPAR-γ expression in articular cartilage. The increased PPAR-γ expression was accompanied by decreased inflammatory responses, which may contribute to the preservation of cartilage integrity.

From a translation perspective, RSGB is an established traditional Chinese medicine formulation that has been developed into an oral liquid preparation with a clinically defined dosage. Liquid formulation inherently offers advantages in gastrointestinal absorption and bioavailability, which is particularly beneficial for elderly KOA patients who often present with compromised metabolic or digestive functions. The dose applied in the present study was determined based on human equivalent dose conversion and further optimized according to preliminary experimental evaluation, providing a rational basis for its application in the KOA model. Although RSGB has been clinically used for other indications, its efficacy in KOA has not yet been established. Therefore, our findings provide preliminary preclinical evidence supporting the potential expansion of RSGB application in KOA management, serving as a promising candidate for drug repurposing. However, further pharmacokinetic studies and well-designed randomized controlled trials are required to validate its efficacy, safety, and optimal dosage in KOA patients. In addition, swimming exercise represents a highly feasible and advantageous non-pharmacological intervention for KOA patients. Due to its low-impact characteristics, aquatic exercise significantly reduces mechanical loading on the knee joint compared with weight-bearing land exercises. The buoyancy of water uniquely counteracts gravity, substantially unloading the vulnerable articular cartilage and subchondral bone, thereby minimizing mechanical friction and pain during movement. Simultaneously, the natural viscosity of water provides uniform, omnidirectional resistance, facilitating the strengthening of peri-articular musculature essential for dynamic knee stabilization, while the hydrostatic pressure aids in reducing joint effusion and edema (Peng et al., 2025). Appropriate aquatic exercise has been widely recommended as a core strategy for individuals with joint pain or functional limitations. Nonetheless, the clinical implementation of this therapy requires a precision medicine approach. Exercise intensity, duration, and frequency must be individually formulated as a customized “exercise prescription.” These prescriptions should be rigorously adjusted according to the patient’s physical capacity, cardiovascular health, and comorbidities to maximize therapeutic benefits without exacerbating local joint inflammation.

Several limitations should be acknowledged in the present study. Although our findings support an association between gut microbiota remodeling and the HDAC3/PPAR-γ signaling pathway during the therapeutic effects of RSGB combined with swimming, the current evidence does not fully establish a causal relationship. In particular, SCFA levels and HDAC enzymatic activity were not directly measured, and further studies involving targeted manipulation of HDAC3/PPAR-γ signaling, quantification of microbiota-derived metabolites, and functional validation are required to clarify the underlying mechanisms. In addition, although the MIA-induced KOA model provides valuable insights into disease progression and therapeutic responses, further studies using larger cohorts and clinical investigations are necessary to validate the efficacy, safety, and translational applicability of RSGB combined with swimming in patients with KOA. In conclusion, the combination of RSGB oral liquid and swimming exercise offers a comprehensive therapeutic strategy for KOA. While swimming primarily contributes to restoring joint motor function and alleviating tactile hypersensitivity, RSGB demonstrates a superior advantage in suppressing systemic pro-inflammatory cytokines to exert its anti-inflammatory and tissue-repairing effects. Furthermore, these different therapeutic modalities exhibit distinct and complementary advantages in specifically modulating targeted gut microbiota populations. Together, they provide enhanced protection against KOA by remodeling the gut microbiota, which sequentially modulates the HDAC3/PPAR-γ signaling axis associated with the gut-joint network to preserve cartilage homeostasis and suppress inflammation. These findings highlight the potential of integrating Traditional Chinese Medicine with targeted exercise prescriptions as a holistic clinical treatment for KOA.

## Conclusion

Combining targeted physical exercise, such as swimming, with pharmacological interventions provides an enhanced therapeutic strategy for KOA that is markedly superior to monotherapy. This comprehensive modality effectively alleviates joint pain and restores articular motor function, while simultaneously preserving cartilage and subchondral bone microarchitecture through the profound suppression of systemic and local inflammation. Mechanistically, altered microbiota composition and altered HDAC3/PPAR-γ expression were associated with the therapeutic effects. Whether regulated by upstream epigenetic factors or mediated through the “gut-joint axis” via the restoration of gut microbiota homeostasis, this combined approach successfully curbs the pathogenic overactivation of HDAC3. The subsequent functional reactivation of the chondroprotective factor PPAR-γ was accompanied by reduced serum NF-κB levels and lower levels of downstream pro-inflammatory cytokines and MMPs. Ultimately, integrating tailored exercise prescriptions with multi-target pharmacological agents establishes a highly promising, holistic clinical paradigm for halting the progression of KOA.

## Data Availability

The raw data supporting the conclusions of this article will be made available by the authors, without undue reservation.

## Footnotes

1
https://cloud.majorbio.com

## Glossary

KOA: Knee osteoarthritis
RSGB: Renshenguben
MIA: Monosodium iodoacetate
SD: Sprague–Dawley
NSAIDs: Nonsteroidal anti-inflammatory drugs
TNF-α: Tumor necrosis factor-α
IL-1β: Interleukin-1β
NF-κB: Nuclear factor kappa-B
MMPs: Matrix metalloproteinases
HDAC3: Histone deacetylase 3
PPAR-γ: Peroxisome proliferator-activated receptor-γ
LPS: Lipopolysaccharide
SCFAs: Short-chain fatty acids
TCM: Traditional Chinese medicine
SPF: Specific pathogen-free
BMD: Bone mineral density
BV/TV: Bone volume fraction
Tb.Th: Trabecular thickness
Tb.Sp: Trabecular separation
SMI: Structural model index
CRP: C-reactive protein
COMP: Cartilage oligomeric matrix protein
IL-10: Interleukin-10
DAO: Diamine oxidase
H&E: Hematoxylin and eosin
Col II: Collagen II
DAB: Diaminobenzidine
ANOVA: One-way analysis of variance
ASVs: Amplicon sequence variants
PCoA: Principal coordinates analysis
NMDS: Non-metric multidimensional scaling
GMHI: Gut microbial health index
MDI: Microbial dysbiosis index

## References

[ref1] Adães S. Mendonça M. Santos T. N. Castro-Lopes J. M. Ferreira-Gomes J. Neto F. L. (2014). Intra-articular injection of collagenase in the knee of rats as an alternative model to study nociception associated with osteoarthritis. Arthritis Res. Ther. 16:R10. doi: 10.1186/ar4436, 24423138 PMC3979102

[ref2] Batushansky A. Zhu S. Komaravolu R. K. South S. Mehta-D’souza P. Griffin T. M. (2022). Fundamentals of OA. An initiative of osteoarthritis and cartilage. Obesity and metabolic factors in OA. Osteoarthr. Cartil. 30, 501–515. doi: 10.1016/j.joca.2021.06.013 PMC8926936 34537381

[ref3] Boileau C. Martel-Pelletier J. Fahmi H. Mineau F. Boily M. Pelletier J. P. (2007). The peroxisome proliferator–activated receptor γ agonist pioglitazone reduces the development of cartilage lesions in an experimental dog model of osteoarthritis: in vivo protective effects mediated through the inhibition of key signaling and catabolic pathways. Arthritis Rheum. 56, 2288–2298. doi: 10.1002/art.22726, 17599749

[ref4] Chan L. C. Li H. H. T. Chan P. K. Wen C. (2021). A machine learning-based approach to decipher multi-etiology of knee osteoarthritis onset and deterioration. Osteoarthritis Cartilage Open 3:100135. doi: 10.1016/j.ocarto.2020.100135, 36475069 PMC9718099

[ref5] Chaplan S. R. Bach F. W. Pogrel J. W. Chung J. M. Yaksh T. L. (1994). Quantitative assessment of tactile allodynia in the rat paw. J. Neurosci. Methods 53, 55–63. doi: 10.1016/0165-0270(94)90144-9, 7990513

[ref6] Chung M. Zhao N. Wong J. B. Wang C. (2016). Prospective economic evaluation of the costs for medications and nutraceuticals in knee osteoarthritis. Osteoarthr. Cartil. 24, S349–S350. doi: 10.1016/j.joca.2016.01.629

[ref7] Cross M. Smith E. Hoy D. Nolte S. Ackerman I. Fransen M. . (2014). The global burden of hip and knee osteoarthritis: estimates from the global burden of disease 2010 study. Ann. Rheum. Dis. 73, 1323–1330. doi: 10.1136/annrheumdis-2013-204763, 24553908

[ref8] de Sire A. Marotta N. Marinaro C. Curci C. Invernizzi M. Ammendolia A. (2021). Role of physical exercise and nutraceuticals in modulating molecular pathways of osteoarthritis. Int. J. Mol. Sci. 22, 5722. doi: 10.3390/ijms22115722, 34072015 PMC8198532

[ref9] Ding L. Zhou J. Ye L. Sun Y. Jiang Z. Gan D. . (2020). PPAR-gamma is critical for HDAC3-mediated control of oligodendrocyte progenitor cell proliferation and differentiation after focal demyelination. Mol. Neurobiol. 57, 4810–4824. doi: 10.1007/s12035-020-02060-8 32803489

[ref10] Elmajee M. Mersal M. Zehra B. Ben Nafa W. Elsayed A. Embaby O. . (2025). Knee osteoarthritis: current insights into pathophysiology and non-surgical management options. Cureus 17:e95302. doi: 10.7759/cureus.95302, 41287699 PMC12640556

[ref11] Fajas L. Egler V. Reiter R. Hansen J. Kristiansen K. Debril M. B. . (2002). The retinoblastoma-histone deacetylase 3 complex inhibits PPARgamma and adipocyte differentiation. Dev. Cell 3, 903–910. doi: 10.1016/s1534-5807(02)00360-x, 12479814

[ref12] Feng Q. Li G. Xia W. Dai G. Zhou J. Xu Y. . (2022). The anti-aging effects of Renshen Guben on thyrotoxicosis mice: improving immunosenescence, hypoproteinemia, lipotoxicity, and intestinal flora. Front. Immunol. 13, 983501. doi: 10.3389/fimmu.2022.983501, 36389720 PMC9640368

[ref13] Gao Z. He Q. Peng B. Chiao P. J. Ye J. (2006). Regulation of nuclear translocation of HDAC3 by IkappaBalpha is required for tumor necrosis factor inhibition of peroxisome proliferator-activated receptor gamma function. J. Biol. Chem. 281, 4540–4547. doi: 10.1074/jbc.M507784200, 16371367 PMC1447600

[ref14] Ghouri A. Conaghan P. G. (2020). Prospects for therapies in osteoarthritis. Calcif. Tissue Int. 109, 339–350. doi: 10.1007/s00223-020-00672-9, 32055890 PMC8403110

[ref15] Gonzalez-Sarrias A. Nunez-Sanchez M. A. Avila-Galvez M. A. Monedero-Saiz T. Rodríguez-Gil F. J. Martínez-Díaz F. . (2018). Consumption of pomegranate decreases plasma lipopolysaccharide-binding protein levels, a marker of metabolic endotoxemia, in patients with newly diagnosed colorectal cancer: a randomized controlled clinical trial. Food Funct. 9, 2617–2622. doi: 10.1039/c8fo00264a, 29770393

[ref16] Gu X. Zhou J. Zhou Y. Wang H. Si N. Ren W. . (2021). Huanglian Jiedu decoction remodels the periphery microenvironment to inhibit Alzheimer’s disease progression based on the “brain-gut” axis through multiple integrated omics. Alzheimer's Res. Ther. 13:44. doi: 10.1186/s13195-021-00779-7, 33579351 PMC7881564

[ref17] Han S. J. Jun J. Eyun S.-i. Lee C.-G. Jeon J. Pan C.-H. (2021). Schisandrol a suppresses catabolic factor expression by blocking NF-κB signaling in osteoarthritis. Pharmaceuticals 14, 241. doi: 10.3390/ph14030241, 33800441 PMC7999623

[ref18] Jiang S. Shen B. (2023). Research progress on the relationship between gut microbiota dysbiosis and osteoarthritis. Zhongguo Xiu Fu Chong Jian Wai Ke Za Zhi 37, 371–376. doi: 10.7507/1002-1892.202212037, 36940999 PMC10027516

[ref19] Kim S. (2008). Changes in surgical loads and economic burden of hip and knee replacements in the US: 1997–2004. Arthritis Care Res. 59, 481–488. doi: 10.1002/art.23525, 18383407

[ref20] Kingkaw A. Raethong N. Patumcharoenpol P. Suratannon N. Nakphaichit M. Keawsompong S. . (2022). Analyzing predominant bacterial species and potential short-chain fatty acid-associated metabolic routes in human gut microbiome using integrative metagenomics. Biology (Basel) 12, 21. doi: 10.3390/biology12010021 PMC9855101 36671714

[ref21] Kobayashi T. Notoya K. Naito T. Unno S. Nakamura A. Martel-Pelletier J. . (2005). Pioglitazone, a peroxisome proliferator–activated receptor γ agonist, reduces the progression of experimental osteoarthritis in guinea pigs. Arthritis Rheum. 52, 479–487. doi: 10.1002/art.20792, 15692987

[ref22] Li S. H. Wu Q. F. (2021). MicroRNAs target on cartilage extracellular matrix degradation of knee osteoarthritis. Eur. Rev. Med. Pharmacol. Sci. 25, 1185–1197. doi: 10.26355/eurrev_202102_24821, 33629288

[ref23] Li X. M. Xu B. Wang Y. P. Wei L. (2014). Anti-inflammatory effect of peroxisome proliferator-activated receptor-γ (PPAR-γ) on non-obese diabetic mice with Sjogren's syndrome. Int J Clin Exp Patho. 7, 4886–4894. PMC4152049 25197359

[ref24] Liu X. Cheng Y. Shao L. Ling Z. (2020). Alterations of the predominant fecal microbiota and disruption of the gut mucosal barrier in patients with early-stage colorectal Cancer. Biomed. Res. Int. 2020:2948282. doi: 10.1155/2020/2948282, 32280686 PMC7114766

[ref25] Loza E. Lopez-Gomez J. M. Abasolo L. Maese J. Carmona L. Batlle-Gualda E. (2009). Economic burden of knee and hip osteoarthritis in Spain. Arthritis Care Res. 61, 158–165. doi: 10.1002/art.24214, 19177521

[ref26] Lu M. Guo S. Nie Z. Ji J. Wang Y. Jiang X. . (2026). Differences in gut microbiota composition are an important reason for lower serum p-cresol sulfate levels in anuric peritoneal dialysis patients compared to hemodialysis patients. Curr. Res. Microbial Sci. 10:100548. doi: 10.1016/j.crmicr.2026.100548, 41568166 PMC12818085

[ref27] Lund H. Weile U. Christensen R. Rostock B. Downey A. Bartels E. M. . (2008). A randomized controlled trial of aquatic and land-based exercise in patients with knee osteoarthritis. J. Rehabil. Med. 40, 137–144. doi: 10.2340/16501977-0134, 18509579

[ref28] Lunz W. Peluzio M. C. G. Dias C. M. G. C. Moreira A. P. B. Natali A. J. (2008). Long-term aerobic swimming training by rats reduces the number of aberrant crypt foci in 1,2-dimethylhydrazine-induced colon cancer. Braz. J. Med. Biol. Res. 41, 1000–1004. doi: 10.1590/s0100-879x2008001100009, 19099153

[ref29] Meng F. Li Z. Zhang Z. Yang Z. Kang Y. Zhao X. . (2018). MicroRNA-193b-3p regulates chondrogenesis and chondrocyte metabolism by targeting HDAC3. Theranostics 8, 2862–2883. doi: 10.7150/thno.23547, 29774080 PMC5957014

[ref30] Morrison D. J. Preston T. (2016). Formation of short chain fatty acids by the gut microbiota and their impact on human metabolism. Gut Microbes 7, 189–200. doi: 10.1080/19490976.2015.1134082, 26963409 PMC4939913

[ref31] Parada-Venegas D. De la Fuente López M. Dubois-Camacho K. Landskron G. Blokzijl T. Molina H. . (2025). Butyrate suppresses mucosal inflammation in inflammatory bowel disease primarily through HDAC3 inhibition in monocytes and macrophages. FEBS J. 292, 6134–6157. doi: 10.1111/febs.70289, 41110099 PMC12631161

[ref32] Park J. Park H. Lee Y. Weon S. Kim Y. G. Yang J. H. . (2021). Blocking TNFα attenuates progressive cartilage matrix degradation in inflammatory arthritis. Exp. Ther. Med. 22:808. doi: 10.3892/etm.2021.10240, 34093764 PMC8170641

[ref33] Pascual G. Fong A. L. Ogawa S. Gamliel A. Li A. C. Perissi V. . (2005). A SUMOylation-dependent pathway mediates transrepression of inflammatory response genes by PPAR-γ. Nature 437, 759–763. doi: 10.1038/nature03988, 16127449 PMC1464798

[ref34] Peng Y. Zou Y. Asakawa T. (2025). The glamor of and insights regarding hydrotherapy, from simple immersion to advanced computer-assisted exercises: a narrative review. Biosci. Trends 19, 10–30. doi: 10.5582/bst.2024.01356, 39756867

[ref35] Saber M. M. Mahmoud M. M. Amin H. M. Essam R. M. (2023). Therapeutic effects of combining curcumin and swimming in osteoarthritis using a rat model. Biomed. Pharmacother. 166:115309. doi: 10.1016/j.biopha.2023.115309, 37573656 PMC10538387

[ref36] Setoguchi K. Misaki Y. Terauchi Y. Yamauchi T. Kawahata K. Kadowaki T. . (2001). Peroxisome proliferator-activated receptor-γ haploinsufficiency enhances B cell proliferative responses and exacerbates experimentally induced arthritis. J. Clin. Invest. 108, 1667–1675. doi: 10.1172/jci13202, 11733562 PMC200985

[ref37] Shumnalieva R. Kotov G. Monov S. (2023). Obesity-related knee osteoarthritis—current concepts. Life 13:13. doi: 10.3390/life13081650, 37629507 PMC10456094

[ref38] Silverwood V. Blagojevic-Bucknall M. Jinks C. Jordan J. L. Protheroe J. Jordan K. P. (2015). Current evidence on risk factors for knee osteoarthritis in older adults: a systematic review and meta-analysis. Osteoarthr. Cartil. 23, 507–515. doi: 10.1016/j.joca.2014.11.019, 25447976

[ref39] Song X. Liu Y. Chen S. Zhang L. Zhang H. Shen X. . (2024). Knee osteoarthritis: a review of animal models and intervention of traditional Chinese medicine. Animal Models Exp. Med. 7, 114–126. doi: 10.1002/ame2.12389, 38409942 PMC11079151

[ref40] Thomas S. Browne H. Mobasheri A. Rayman M. P. (2018). What is the evidence for a role for diet and nutrition in osteoarthritis? Rheumatology 57, iv61–iv74. doi: 10.1093/rheumatology/key011, 29684218 PMC5905611

[ref41] Vasheghani F. Monemdjou R. Fahmi H. Zhang Y. Perez G. Blati M. . (2013). Adult cartilage-specific peroxisome proliferator–activated receptor gamma knockout mice exhibit the spontaneous osteoarthritis phenotype. Am. J. Pathol. 182, 1099–1106. doi: 10.1016/j.ajpath.2012.12.012 23375622

[ref42] Vasheghani F. Zhang Y. Li Y.-H. Blati M. Fahmi H. Lussier B. . (2015). PPARγ deficiency results in severe, accelerated osteoarthritis associated with aberrant mTOR signalling in the articular cartilage. Ann. Rheum. Dis. 74, 569–578. doi: 10.1136/annrheumdis-2014-205743, 25573665 PMC4345902

[ref43] Vighi G. Marcucci F. Sensi L. Di Cara G. Frati F. (2008). Allergy and the gastrointestinal system. Clin. Exp. Immunol. 153, 3–6. doi: 10.1111/j.1365-2249.2008.03713.x, 18721321 PMC2515351

[ref44] Vonsy J. L. Ghandehari J. Dickenson A. H. (2012). Differential analgesic effects of morphine and gabapentin on behavioural measures of pain and disability in a model of osteoarthritis pain in rats. Eur. J. Pain 13, 786–793. doi: 10.1016/j.ejpain.2008.09.008, 18955000

[ref45] Wilson A. J. Byun D.-S. Popova N. Murray L. B. L'Italien K. Sowa Y. . (2006). Histone deacetylase 3 (HDAC3) and other class I HDACs regulate Colon cell maturation and p21 expression and are deregulated in human Colon Cancer. J. Biol. Chem. 281, 13548–13558. doi: 10.1074/jbc.M510023200, 16533812

[ref46] Wojcieszek A. Kurowska A. Majda A. Liszka H. Gądek A. (2022). The impact of chronic pain, stiffness and difficulties in performing daily activities on the quality of life of older patients with knee osteoarthritis. Int. J. Environ. Res. Public Health 19, 16815. doi: 10.3390/ijerph192416815, 36554695 PMC9779661

[ref47] Xu L. Ma J. Zhou C. Shen Z. Zhu K. Wu X. (2024). Identification of key hub genes in knee osteoarthritis through integrated bioinformatics analysis. Sci. Rep. 14:22437. doi: 10.1038/s41598-024-73188-z PMC11439059 39341952

[ref48] Xu H. Xu B. (2021). BMSC-derived exosomes ameliorate osteoarthritis by inhibiting pyroptosis of cartilage via delivering miR-326 targeting HDAC3 and STAT1//NF-κB p65 to chondrocytes. Mediat. Inflamm. 9972805. doi: 10.1155/2021/9972805 PMC8577926 34764819

[ref49] Zeng L. Zhou G. Yang W. Liu J. (2023). Guidelines for the diagnosis and treatment of knee osteoarthritis with integrative medicine based on traditional Chinese medicine. Front. Med. 10, 1260943. doi: 10.3389/fmed.2023.1260943, 37915321 PMC10617515

[ref50] Zhang J. Peng J. Zhang T. Jiang H. Qin Y. Chen H. . (2023). Identification of the Main chemical constituents and mechanism of Renshen Guben oral liquid against renal fibrosis. Chin. Med. 18:56. doi: 10.1186/s13020-023-00762-4, 37198665 PMC10190030

[ref51] Zhou Q. Liu J. Wan L. Zhu Y. Qi Y. Hu Y. (2024). Xinfeng capsule alleviates interleukin-1beta-induced chondrocyte inflammation and extracellular matrix degradation by regulating the miR-502-5p/TRAF2/NF-kappaB axis. Nan Fang Yi Ke Da Xue Xue Bao 44, 108–118. doi: 10.12122/j.issn.1673-4254.2024.01.13, 38293982 PMC10878885

[ref52] Zhu X. Chen F. Lu K. Wei A. Jiang Q. Cao W. (2019). PPARγ preservation via promoter demethylation alleviates osteoarthritis in mice. Ann. Rheum. Dis. 78, 1420–1429. doi: 10.1136/annrheumdis-2018-214940, 31239244

[ref53] Zhu J. K. He T. D. Wei Z. X. Wang Y. M. (2018). LncRNA FAS-AS1 promotes the degradation of extracellular matrix of cartilage in osteoarthritis. Eur. Rev. Med. Pharmacol. Sci. 22, 2966–2972. doi: 10.26355/eurrev_201805_15051, 29863238

